# Association between serum lipid-apolipoprotein alterations and soluble syndecan-1 dynamics in sepsis: impact of apolipoprotein E isoforms

**DOI:** 10.1016/j.bbrep.2026.102634

**Published:** 2026-05-14

**Authors:** Riho Shimizu, Shogo Akahane, Harue Suzuki, Hiroto Matsuura, Yui Uematsu, Wakana Iimura, Nau Ishimine, Kazuyoshi Yamauchi

**Affiliations:** a Department of Medical Sciences, Graduate School of Medicine, Science and Technology, Shinshu University, Matsumoto, Japan; b Department of Laboratory Medicine, Shinshu University Hospital, Matsumoto, Japan; c Department of Clinical Laboratory, Ina Central Hospital, Ina, Japan; d Department of Biomedical Laboratory Sciences, School of Health Sciences, Shinshu University, Matsumoto, Japan; e Division of NeuroHealth Innovation, Institute for Biomedical Sciences, Shinshu University, Matsumoto, Japan

**Keywords:** Inflammation, Shedding, Dyslipidemia, apoE phenotype, HDL-C, Triglyceride-rich lipoprotein

## Abstract

**Background:**

Dyslipidemia is a common feature of sepsis; however, its relationship with inflammation-related biomarkers remains incompletely understood. This study aimed to examine the associations between soluble syndecan-1 (sSDC1) dynamics and lipid/apolipoprotein profiles in sepsis, focusing on the impact of apolipoprotein (apo) E isoforms.

**Methods:**

Serum levels of sSDC1, lipids, and apolipoproteins were measured and compared between patients with sepsis (n = 259) and non-septic controls (n = 57) at Shinshu University Hospital, Matsumoto, Japan.

**Results:**

Compared to non-septic controls, patients with sepsis exhibited a significant elevation in serum sSDC1 levels and marked dyslipidemia, characterized by decreased levels of high-density lipoprotein cholesterol (HDL-C), low-density lipoprotein cholesterol (LDL-C), apoAI, and apoAII, and increased apoE-containing triglyceride (TG)-rich lipoprotein. The apoE phenotype influences the relationship between sSDC1 levels and lipid/apolipoprotein profiles. Correlation analysis revealed a negative correlation between sSDC1 and HDL-C or apoAI in patients with the apoE2/E3. In contrast, a similar negative correlation between sSDC1 and LDL-C or apoB was observed in those with apoE3/E4. Additionally, a positive correlation between sSDC1 and apoCII was found in patients with apoE2/E3, whereas negative correlations between apoCIII and apoE were observed in those with apoE3/E4. Multivariate analysis showed that serum HDL-C and apoAI levels may be negative predictors of sSDC1 levels, particularly in patients with apoE2/E3. Conversely, apoCIII and TG levels may be positive predictors of sSDC1 levels in patients, particularly in those with apoE2/E3.

**Conclusions:**

Serum sSDC1 levels were associated with alterations in lipid and apolipoprotein profiles in sepsis, and these associations differed according to apoE phenotype.

## Highlights

• Septic patients show significantly elevated serum sSDC1 levels and marked dyslipidemia.
• Sepsis-related dyslipidemia is characterized by reduced HDL-C and altered triglyceride-rich lipoprotein profiles.
• The relationships between sSDC1 levels and lipid/apolipoprotein profiles differ according to apoE phenotype in septic patients.
• ApoE phenotype may influence the association between inflammation-related sSDC1 dynamics and lipid/apolipoprotein alterations in sepsis.

## 1 Introduction

Sepsis is a life-threatening clinical syndrome characterized by organ dysfunction owing to a dysregulated inflammatory response to infection, potentially progressing to septic shock [1]. Lipoprotein metabolism, particularly microbial immunity, is closely associated with the pathophysiology of sepsis [2].

Lipopolysaccharide (LPS)-induced inflammation often leads to lipid metabolism abnormalities, characterized by hypertriglyceridemia and reduced high-density lipoprotein cholesterol (HDL-C) levels [2,3]. Sepsis-related dyslipidemia, including alterations in triglyceride-rich lipoproteins, has been associated with poor clinical outcomes [[2], [3], [4]]. In addition, inflammatory conditions may alter the composition and biological properties of lipoproteins, making the interpretation of dyslipidemia in sepsis more complex [5]. Reduced HDL-C levels have also been reported in sepsis [2,[6], [7], [8]]. However, the relationships between inflammation-related changes and lipid/apolipoprotein abnormalities in sepsis remain incompletely understood.

TRL, such as chylomicrons and very low-density lipoproteins (VLDL), are primarily catabolized through their interaction with the low-density lipoprotein (LDL) receptor (LDLR) [9]. Alternatively, these lipoproteins can be metabolized via a pathway involving the cooperative action of LDLR-related protein (LRP) 1 and syndecan (SDC) 1, which serve as a backup for the LDLR pathway, as demonstrated in both mice and humans [**[**[10], [11], [12], [13]]. Unlike LDLR, this alternative pathway recognizes only apolipoprotein (apo) E and not apoB-100, which is embedded in lipoprotein particles as a ligand [10,11]. We recently reported that the extracellular interaction between apoE-containing TRL (apoE-TRL) and LRP1 is pivotal in regulating the LPS-induced inflammatory response in human fibroblasts [14]. However, in the metabolism of TRL remnants via the LRP1-SDC1 pathway, SDC1, rather than LRP1, is considered the key mediator [12].

SDC1, a type I transmembrane heparan sulfate proteoglycan, is expressed on the cell surface and in the extracellular matrix of mammalian cells, particularly epithelial cells [15,16], and its core protein comprises extracellular, transmembrane, and intracellular domains [17]. In addition to its role in TRL metabolism, SDC1 contributes to various biological processes, including regulating diverse cell behaviors (e.g., migration, adhesion, and invasion) and signal transduction [[17], [18], [19]]. The ectodomain of SDC1 is proteolytically shed from the cell surface and released into the bloodstream as soluble SDC1 (sSDC1). SDC1 shedding is enhanced in various pathological conditions, including inflammation, tumor progression, and wound healing, and regulates these processes in both preclinical studies using rodent models and clinical studies [[20], [21], [22]]. Pathologically enhanced SDC1 shedding leads to elevated serum sSDC1 levels, making it a promising biomarker for various diseases.

Human apoE, a 34-kDa glycoprotein, has three major isoforms (apoE2, apoE3, and apoE4) encoded by three independent alleles at a single locus [23]. Like SDC1 and LRP1, apoE is essential for lipid homeostasis and immunoregulation [23,24]. In rodent models of LPS-induced inflammation, apoE attenuates the host inflammatory response and protects against mortality [25,26]. ApoE isoforms differ in their binding affinities for LDLR, influencing apoE-TRL metabolism [23]. Several studies, including those using human apoE knock-in mice and other relevant experimental models [27], as well as isogenic iPSC-derived astrocytes [28], have suggested that the anti-inflammatory ability of apoE varies among its isoforms; while apoE2 and apoE3 show anti-inflammatory effects, apoE4 shows a proinflammatory effect [27,28]. However, the precise inflammatory role of apoE isoforms remains unclear.

Based on previous evidence suggesting that the SDC1-LRP1 pathway is involved in apoE-containing lipoprotein metabolism and inflammatory regulation, we hypothesized that serum sSDC1 levels would be associated with lipid/apolipoprotein profiles in septic patients, and that these associations might differ according to apoE phenotype. The present study aimed to explore the clinical associations between serum sSDC1 dynamics and lipid/apolipoprotein profiles in septic patients, with a particular focus on the impact of apoE isoforms.

## 2 Materials and methods

### 2.1 Subjects

This study included 316 Japanese patients enrolled between April 2023 and March 2025 at Shinshu University Hospital (Matsumoto, Japan). The sepsis group consisted of 259 hospitalized patients diagnosed according to the Sepsis-3 criteria [1]. As a control group, 57 outpatients clinically assessed as free of acute inflammatory or severe metabolic disorders were included. To ensure the rigor of the control group, all control subjects were confirmed to exhibit normal results in the following laboratory tests: CRP concentration (<0.05 mg/dL); creatinine concentration (males, 0.65–1.07 mg/dL; females, 0.46–0.79 mg/dL); AST activity (13–30 U/L); ALT activity (males, 10–42 U/L; females, 7–23 U/L); LD activity (124–222 U/L); LDL-C concentration (65–120 mg/dL); HDL-C concentration (50–90 mg/dL); and TG concentration (40–150 mg/dL). All serum samples were aliquoted and stored at −80 °C until analysis. Due to the retrospective nature of the study design, comprehensive clinical documentation was not consistently available. Specifically, data regarding obesity status, sepsis severity scores, length of hospital stay, survival outcomes, infection sites, specific organ dysfunctions (e.g., acute kidney injury and acute lung injury), and mechanical ventilation usage were incomplete for a subset of patients. Available data are summarized in Table 1. Regarding sample collection, to ensure clinical relevance and reflect the acute phase of the condition, only serum samples obtained at the time of or immediately following the diagnosis of sepsis were selected for analysis. The study was conducted in accordance with the Declaration of Helsinki and approved by the Ethical Review Board of Shinshu University School of Medicine (approval number: 5762). Written informed consent was obtained from all patients.

**Table 1:** Baseline characteristics of the study population.

Variable	Sepsis group (n = 259)	Control group (n = 57)
Demographic characteristics		
Age (SD), years	66.8 (15.2)	65.1 (14.7)
Gender, %male	148 (57.1)	25 (43.9)

Clinical outcomes		
Mortality, n (%) a	28 (16.6)	N/A
SOFA score, median (IQR)	7 (5 – 8)	N/A
Septic shock, n (%)	112 (43.2)	N/A
DIC, n (%) b	38 (18.7)	N/A

Underlying diseases		
Diabetes mellitus, n (%)	61 (23.6)	N/A
Dyslipidemia, n (%)	18 (6.9)	N/A
Malignancy, n (%)	70 (27)	N/A

Inflammatory markers		
C-reactive protein (SD), mg/dL	10. 336 (9.823)	0.042 (0.036)
WBC count (SD), × 10^3^/μL	10.07 (6.62)	5.45 (1.63)
Procalcitonin (IQR), ng/mL c	5.69 (1.27 – 15.17)	N/A

Renal function		
Creatinine (SD), mg/dL	1.482 (1.510)	0.746 (0.135)
Acute kidney injury, n (%)	22 (8.5)	N/A
Chronic kidney disease, n (%)	17 (6.6)	N/A

*Infection site*^d^		
Biliary tract, n (%)	55 (29.7)	N/A
Lung, n (%)	49 (26.5)	N/A
Urinary tract, n (%)	45 (24.3)	N/A
Other, n (%) e	36 (19.5)	N/A

*Causative pathogens*^f^		
Gram-negative bacteria, n (%)	124 (60.2)	N/A
Gram-positive bacteria, n (%)	39 (18.9)	N/A
Fungi, n (%)	4 (1.9)	N/A
Mixed (Polymicrobial), n (%)	30 (14.6)	N/A
Unknown, n (%)	9 (4.4)	N/A

Data are presented as mean (SD), median (IQR), or n (%), as appropriate. Abbreviations: SOFA, Sequential Organ Failure Assessment; DIC, disseminated intravascular coagulation; WBC, white blood cell; IQR, interquartile range; SD, standard deviation; N/A, not applicable.

a Mortality data were available for 169 patients.

b DIC was diagnosed in 203 patients.

c Procalcitonin data were available for 206 patients.

d Infection site data available for 185 patients.

e Other infection sites include septic arthritis (n = 19), myocarditis (n = 4), liver abscess (n = 5), gastrointestinal perforation (n = 3), burn injury (n = 3), and cellulitis (n = 1).

f Mixed infections included Gram-positive + Gram-negative (n = 23), Gram-negative + fungi (n = 4), and Gram-negative + COVID-19 (n = 3).

### 2.2 Determination of serum sSDC1 levels

Serum sSDC1 levels were determined using a commercial ELISA kit (Diaclone SAS; Besancon Cedex, France) according to the manufacturer's instructions and our previous report [29]. Briefly, 100 μL/well of standards, controls, and serum samples were added in duplicate to antibody-coated microplate wells, followed by 50 μL/well of biotinylated anti-CD138 antibody. After incubation, washing, and sequential addition of 100 μL/well of streptavidin-HRP and TMB substrate, the reaction was stopped with 100 μL/well of H_2_SO_4_ stop reagent. Absorbance was measured at 450 nm with 620 nm as the reference wavelength, when applicable, and concentrations were calculated from a freshly prepared standard curve.

### 2.3 Determination of serum lipid and apolipoprotein levels

Serum HDL-C and LDL-C levels were measured using homogeneous assay kits (Sekisui Medical Co., Tokyo, Japan). Serum TG levels were determined using the glycerol kinase-glycerol-3-phosphate-peroxidase method (Shino-Test Co., Tokyo, Japan). Serum apolipoprotein (AI, AII, B, CII, CIII, and E) levels were determined by a turbidimetric immunoassay using a commercially available kit (Sekisui Medical Co.). All measurements were performed using a JCA-ZS050 automated analyzer (JEOL, Tokyo, Japan).

### 2.4 ApoE phenotyping

The serum apoE phenotype was determined using isoelectric focusing and immunoblot analysis, as described previously [30]. Briefly, 20 μL of neuraminidase-treated serum was electrophoresed on a 4.8% polyacrylamide gel containing 8 mol/L urea and 20 g/L ampholytes (pH 4–6; Thermo Fisher Scientific Inc., Waltham, MA, USA), using 3.3 mmol/L phosphoric acid as the anode solution and 20 mmol/L NaOH as the cathode solution. Electrophoresis was carried out overnight at 4 °C under a constant voltage of 200 V. After electrophoresis, apoE bands were detected by immunoblot analysis using a horseradish peroxidase-conjugated goat anti-apoE polyclonal antibody (Fortis Life Sciences, Boston, MA, USA).

### 2.5 Statistical methods

Parametric data are presented as means and standard deviations (SD), and nonparametric data as medians and interquartile ranges (IQR). Correlations were evaluated using Spearman's rank correlation coefficient. Differences between the two groups were assessed using the Mann–Whitney *U* test. Results from three or more groups were analyzed using the Kruskal–Wallis test with the Steel–Dwass post hoc test. Allele frequency comparisons were conducted using the chi-squared test or Fisher's exact test, as appropriate. Multivariate regression analysis was performed to identify the independent factors affecting serum sSDC1 levels, with *p* values *<* 0.05 considered significant. All statistical analyses were performed using BellCurve for Excel (Social Survey Research Information Co., Ltd., Tokyo, Japan).

## 3 Results

### 3.1 Patient characteristics

The baseline characteristics of the study population are summarized in Table 1. The study included 259 patients with sepsis and 57 non-septic controls. The mean age was 66.8 ± 15.2 years in the sepsis group and 65.1 ± 14.7 years in the control group, and males accounted for 57.1% and 43.9% of the subjects, respectively. In the sepsis group, the median SOFA score was 7 (IQR, 5–8), 112 patients (43.2%) had septic shock, and 28 of 169 patients with available outcome data (16.6%) died. Diabetes mellitus, dyslipidemia, and malignancy were present in 23.6%, 6.9%, and 27.0% of the patients with sepsis, respectively. Inflammatory and renal function markers, as well as infection site and causative pathogen data, are also summarized. Because of the retrospective design, several clinical variables were available only for subsets of patients.

### 3.2 Serum sSDC1 levels and lipid/apolipoprotein profiles in patients with sepsis

**Fig. 1:**
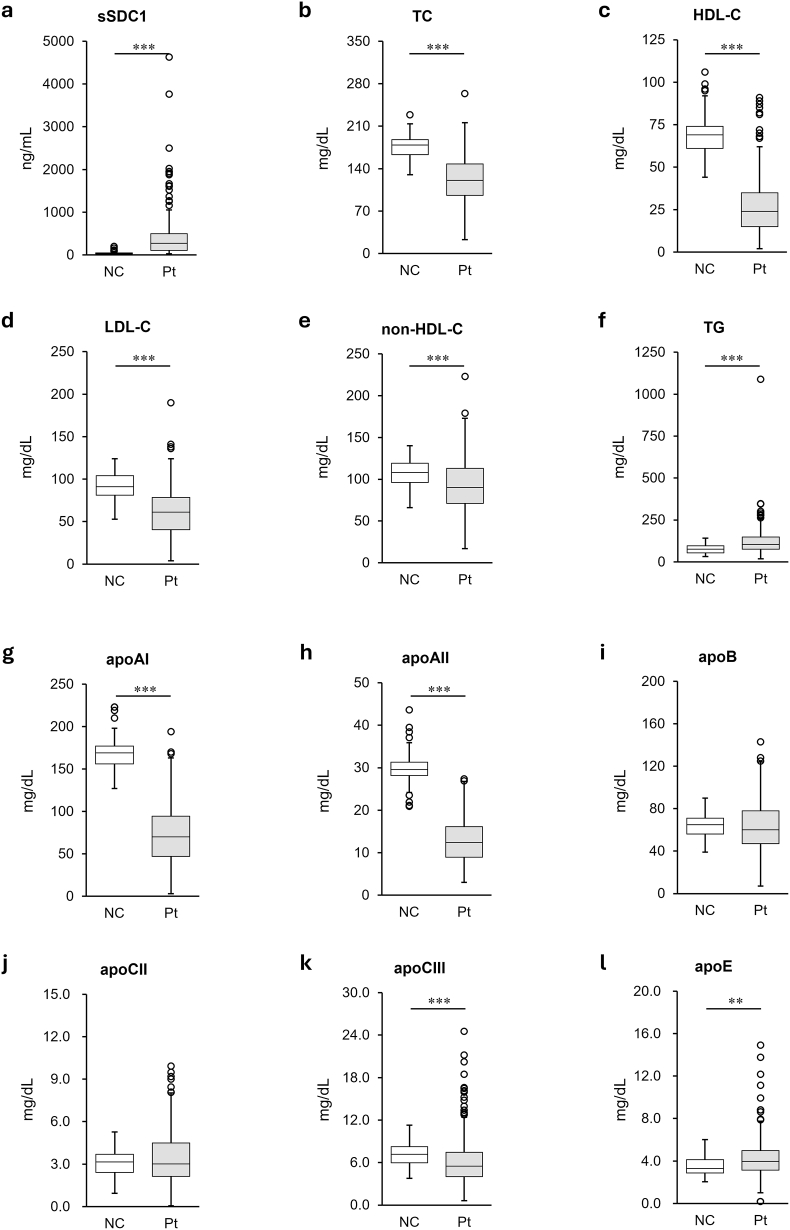
Comparison of serum syndecan-1 (sSDC1) and lipid/apolipoprotein levels in patients with and without sepsis The serum levels of sSDC1 (**a**), total cholesterol (TC) (**b**), high-density lipoprotein cholesterol (HDL-C) (**c**), low-density lipoprotein cholesterol (LDL-C) (**d**), non-HDL-C (**e**), triglyceride (TG) (f), apoAI (**g**), apoAII (**h**), apoB (**i**), apoCII (**j**), apoCIII (**k**), and apoE (**l**) were compared between non-septic control subjects (**NC**; n = 57) and septic patients (**Pt**; n = 259) using the Mann–Whitney *U* test. The data are expressed as box plots. The ends of the bars indicate 25th and 75th percentiles. ∗∗∗*p* < 0.001 and ∗∗*p* < 0.01.

Initially, serum sSDC1 levels and lipid/apolipoprotein profiles were compared between patients with sepsis and control subjects (Fig. 1). Serum sSDC1 levels were significantly higher in patients (274 [108‒495] ng/mL) compared to controls (42 [30‒501] ng/mL) (*p* < 0.001) (Fig. 1a). Additionally, all cholesterol fractions were significantly lower in patients than in controls (TC, 122 ± 37.0 mg/dL vs 175 ± 21.3 mg/dL [*p* < 0.001]) (Fig. 1b); HDL-C, 27.1 ± 17.50 mg/dL vs 68.4 ± 12.51 mg/dL (*p* < 0.001) (Fig. 1c); LDL-C, 61.1 ± 27.92 mg/dL vs 91.4 ± 17.29 mg/dL (*p* < 0.001) (Fig. 1d); non-HDL-C, 95 ± 31.8 mg/dL vs 107 ± 17.6 mg/dL (*p* < 0.001) (Fig. 1e)]. In contrast, TG levels were significantly higher in patients (124 ± 85.6 mg/dL) than in controls (75 ± 826.4 mg/dL) (*p* < 0.001) (Fig. 1f).

For serum apolipoprotein profiles, apoAI and apoAII levels were significantly lower in patients than in controls (apoAI, 72 ± 34.7 mg/dL vs 167 ± 20.9 mg/dL [*p* < 0.001]) (Fig. 1g); apoAII, 12.7 ± 5.04 mg/dL vs 29.9 ± 4.14 mg/dL (*p* < 0.001) (Fig. 1h)]. In contrast, apoE levels were significantly higher in patients (4.3 ± 1.91 mg/dL) than in controls (3.6 ± 0.99 mg/dL) (*p* < 0.001) (Fig. 1i). Additionally, apoCIII levels were significantly lower in patients (6.2 ± 3.57 mg/dL) than in controls (7.3 ± 1.80 mg/dL) (*p* < 0.001) (Fig. 1k). No significant differences in apoB and apoCII levels were observed between the patients and controls (Fig. 1i and j).

### 3.3 Effect of sepsis on the relationship between serum sSDC1 levels and lipid/apolipoprotein profiles

**Fig. 2:**
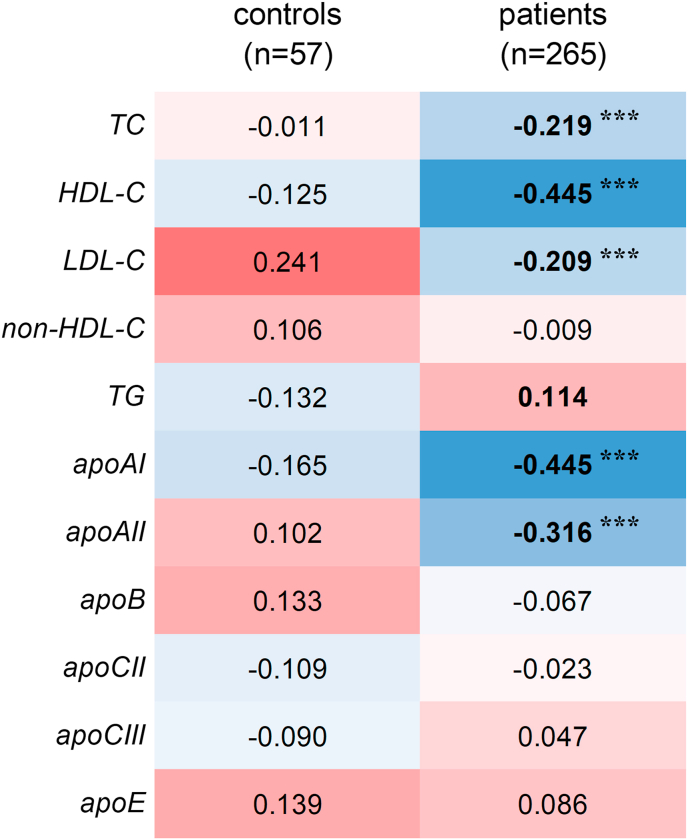
Heatmap of Spearman's correlation between serum sSDC1 and lipid/apolipoprotein levels in patients with and without sepsis Spearman's rank correlation coefficients (ρ) between serum sSDC1 and lipid/apolipoprotein levels in non-septic control subjects and septic patients are shown in each cell of the correlation matrix. Blue and red indicate negative and positive correlations, respectively, with the color intensity corresponding to the strength of the correlation. ∗∗∗*p* < 0.001.

To assess the effect of systemic inflammation on the relationship between SDC1 shedding and lipid metabolism, correlations between serum sSDC1 and lipid/apolipoprotein levels were analyzed in patients with sepsis and control subjects. The results are presented in a heat map (Fig. 2).

In patients, the sSDC1 levels showed a significant negative correlation with the levels of TC (ρ = −0.219, *p* < 0.001), HDL-C (ρ = −0.445, *p* < 0.001), and LDL-C (ρ = −0.209, *p* < 0.001). Notably, the correlation between sSDC1 and LDL-C levels in patients was opposite to that observed in the controls, which showed a weak but positive correlation (ρ = 0.241). In contrast, although no statistically significant difference was observed, the correlation between sSDC1 and TG levels showed a trend toward a positive correlation in patients (ρ = 0.114, *p* = 0.066). For serum apolipoprotein composition, sSDC1 levels in patients showed a significant negative correlation with the levels of apoAI (ρ = −0.445, *p* < 0.001) and apoAII (ρ = −0.316, *p* < 0.001).

### 3.4 Allele frequency of apoE in patients with sepsis

To investigate the association between apoE phenotypes and susceptibility to sepsis, the apoE allele frequencies were compared between patients with sepsis and controls. The frequency of the ε4 allele, which encodes apoE4, was significantly higher in patients compared to controls (odds ratio [OR], 3.779; 95% confidence interval [CI], 1.156–12.354; *p* = 0.019) (Table 2).

**Table 2:** Allele frequency of apoE in patients with sepsis.

apoE allele	allele frequency		OR	95%CI	*p* value
	sepsis	control			
ε2	35	9	0.845	0.395 – 1.812	0.666
ε3	435	102	0.611	0.324 – 1.172	0.137
ε4	48	3	3.779	1.156 – 12.354	0.019∗

OR, odds ratio; CI, confidence interval. ∗, *p* < 0.05.

### 3.5 Effect of apoE phenotype on serum lipid/apolipoprotein profiles in patients with sepsis

**Fig. 3:**
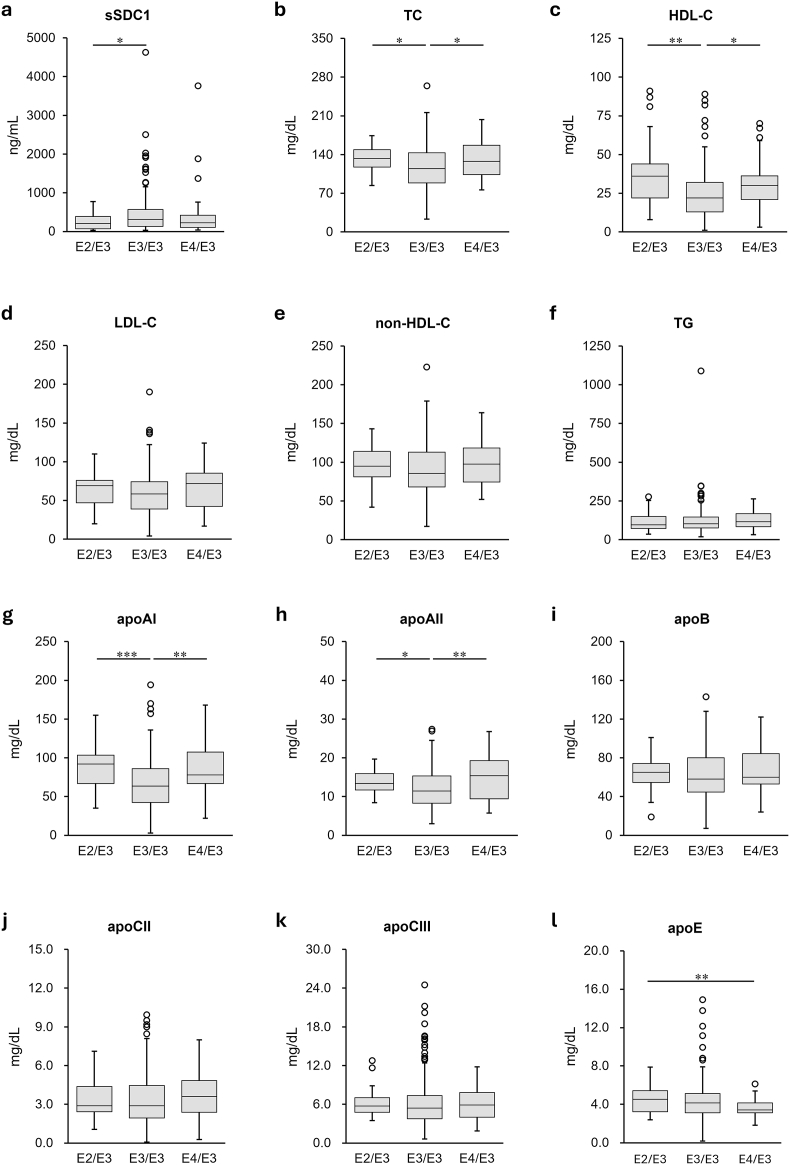
Comparison of serum sSDC1 and lipid/apolipoprotein levels among apoE phenotype groups in septic patients The serum levels of sSDC1 (**a**), TC (**b**), HDL-C (**c**), LDL-C (**d**), non-HDL-C (**e**), TG (f), apoAI (**g**), apoAII (**h**), apoB (**i**), apoCII (**j**), apoCIII (**k**), and apoE (**l**) were compared among the apoE phenotype groups (apoE2/E3, n = 35; apoE3/E3, n = 176; apoE3/E4, n = 48) using the Kruskal–Wallis test with the Steel–Dwass post-hoc test. The data are expressed as box plots. The ends of the bars indicate 25th and 75th percentiles. ∗∗∗*p* < 0.001, ∗∗*p* < 0.01, and ∗*p* < 0.05.

Serum sSDC1 levels in patients with apoE3/E3 were significantly higher (313 [133‒563] ng/mL) than in those with apoE2/E3 (201 [68-384] ng/mL) (*p* < 0.05). Additionally, although no statistical significance was observed, sSDC1 levels in patients with apoE3/E3 tended to be higher than those in patients with apoE4/E3 (224 [104‒419] ng/mL) (Fig. 3a).

The levels of TC and HDL-C were significantly lower in patients with apoE3/E3 (TC, 118 ± 40.0 mg/dL; HDL-C, 24.7 ± 16.18 mg/dL) compared to those in patients with apoE2/E3 (TC, 132 ± 21.8 mg/dL [*p* < 0.05]; HDL-C, 36.7 ± 21.06 mg/dL [*p* < 0.01]) and apoE4/E3 (TC, 132 ± 33.9 mg/dL [*p* < 0.05]; HDL-C, 31.4 ± 16.42 mg/dL [*p* < 0.05]) (Fig. 3b and c). No differences were observed in the LDL-C, non-HDL-C, or TG levels among the three apoE phenotypes (Fig. 3d, e, and f).

Similarly, the levels of apoAI and apoAII were significantly lower in patients with apoE3/E3 (apoAI 66 ± 33.9 mg/dL; apoAII, 12.1 ± 5.09 mg/dL) compared to those in patients with apoE2/E3 (apoAI, 88 ± 29.3 mg/dL [*p* < 0.001]; apoAII, 13.7 ± 3.05 mg/dL[*p* < 0.05]) and apoE4/E3 (apoAI, 86 ± 34.2 mg/dL [*p* < 0.005]; apoAII, 14.8 ± 5.53 mg/dL[*p* < 0.01]) (Fig. 3g and h). On the contrary, apoE levels were significantly higher in patients with apoE2/E3 (4.5 ± 1.30 mg/dL) compared to those in patients with apoE4/E3 (3.6 ± 0.91 mg/dL [*p* < 0.01]). Although not statistically significant, apoE levels in patients with apoE3/E3 (4.4 ± 2.06 mg/dL) tended to be higher than in patients with apoE4/E3 (Fig. 3l). No significant differences were observed in the apoB, apoCII, or apoCIII levels among the three apoE phenotypes (Fig. 3i, j, and k).

### 3.6 Effect of apoE phenotype on the relationship between serum SDC1 levels and lipid/apolipoprotein profiles in patients with sepsis

**Fig. 4:**
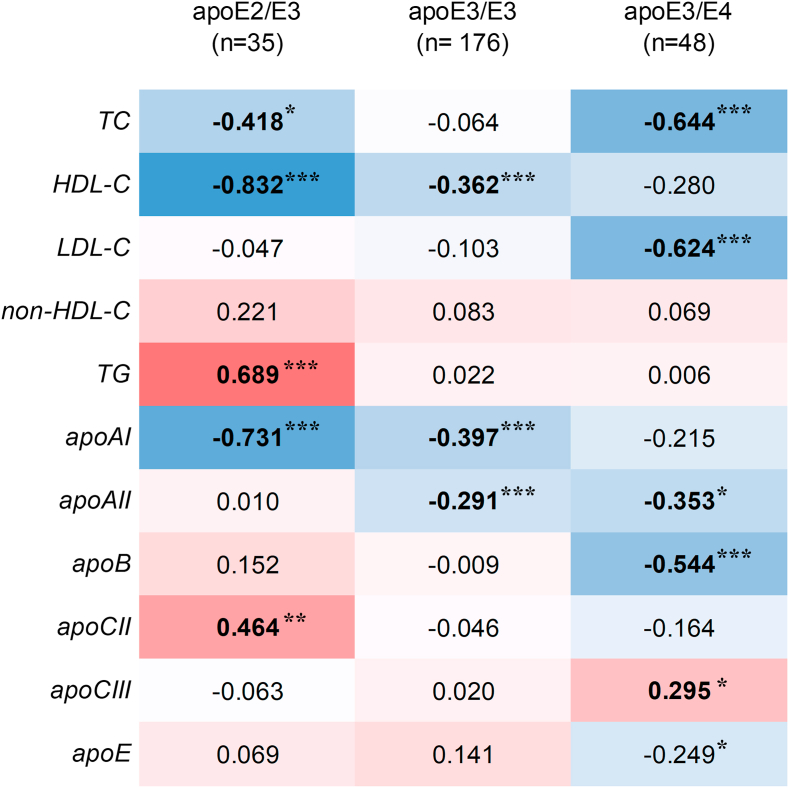
Heatmap of Spearman's correlation between serum sSDC1 and lipid/apolipoprotein levels among apoE phenotype groups in septic patients Spearman's rank correlation coefficients (ρ) between serum sSDC1 and lipid/apolipoprotein levels in septic patients, stratified by apoE phenotype, are shown in each cell of the correlation matrix. Blue and red indicate negative and positive correlations, respectively, with the color intensity corresponding to the strength of the correlation. ∗∗∗*p* < 0.001, ∗∗*p* < 0.01, and ∗*p* < 0.05.

To further assess the effect of the apoE phenotype on the relationship between SDC1 shedding and lipid metabolism, correlations between serum sSDC1 levels and lipid/apolipoprotein levels were analyzed in patients with sepsis among the three apoE phenotypes. The results are presented in a heat map (Fig. 4).

The negative correlation between sSDC1 and TC in patients was more pronounced in patients with apoE3/E4 (ρ = −0.644, *p* < 0.001) than in those with apoE2/E3 (ρ = −0.418 *p* < 0.01), but no such correlation was observed in those with apoE3/E3 (ρ = −0.064). In contrast, a negative correlation between sSDC1 and HDL-C was observed across all phenotypes, including apoE3/E3 (ρ = −0.362, *p* < 0.001), and was most pronounced in those with apoE2/E3 (ρ = −0.832, *p* < 0.001). However, this correlation was not statistically significant in those with apoE3/E4 (ρ = −0.280). The negative correlation between sSDC1 and LDL-C was specific to patients with the apoE3/E4 phenotype (ρ = −0.624, *p* < 0.001), and no such correlation was observed in those with the apoE2/E3 (ρ = −0.28047) or apoE3/E3 (ρ = −0.103) phenotypes. Finally, a positive correlation between sSDC1 and TGs was observed specifically in patients with the apoE2/E3 phenotype (ρ = 0.689, *p* < 0.001).

For serum apolipoprotein profiles, the strength of the negative correlation between sSDC1 and apoAI in patients followed the order: apoE2/E3 (ρ = −0.731, *p* < 0.001), apoE3/E3(ρ = −0.397, *p* < 0.001), and apoE3/E4(ρ = −0.215). In contrast, the negative correlation between sSDC1 and apoAII was observed in patients with apoE3/E3 (ρ = −0.291, *p* < 0.001) and apoE3/E4 (ρ = −0.397, *p* < 0.05), but not in those with apoE2/E3 (ρ = 0.010). No correlation was observed between sSDC1 and apoB, apoCII, or apoCIII levels in any patient. However, when the apoE phenotype was analyzed, significant correlations were observed. Specifically, in patients with apoE3/E4, a negative correlation was observed between sSDC1 and apoB (ρ = −0.545, *p* < 0.001), while a positive correlation was found between sSDC1 and apoCIII (ρ = 0.295, *p* < 0.05). In patients with apoE2/E3, a positive correlation was observed between sSDC1 and apoCII (ρ = 0.464, *p* < 0.001). The negative correlation between sSDC1 and apoE was specifically observed in patients with apoE3/E4 (ρ = −0.249, *p* < 0.05).

### 3.7 Determination of the factors affecting serum sSDC1 levels in patients with sepsis

**Fig. 5:**
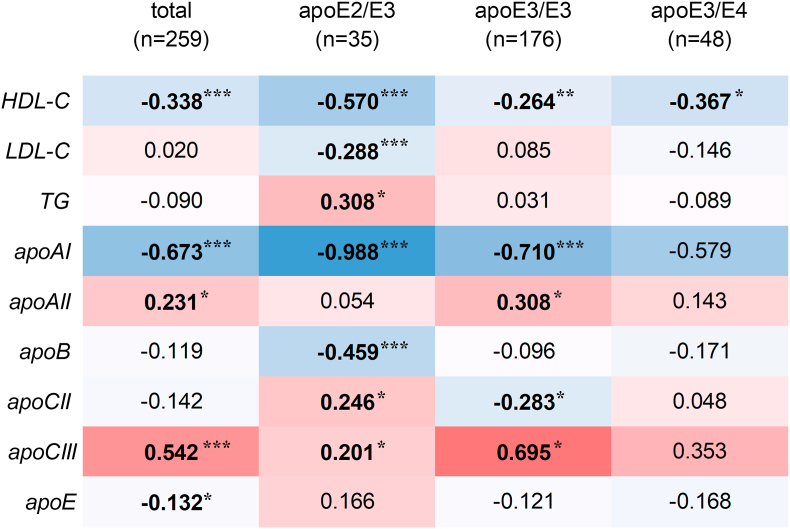
**Heatmap of standardized partial regression coefficient for predictors of serum sSDC1 levels in septic patients among the apoE phenotype group** The standardized partial regression coefficient (β) between serum sSDC1 and lipid/apolipoprotein levels in patients with sepsis stratified by the apoE phenotype is shown in each cell of the correlation matrix. Blue and red indicate the negative and positive effects on serum sSDC1 levels, respectively, with the color intensity corresponding to the strength of the correlation. ∗∗∗*p* < 0.001, ∗∗*p* < 0.01, and ∗*p* < 0.05.

To determine the factors affecting serum sSDC1 levels in patients with sepsis, a multiple regression analysis was conducted with serum sSDC1 levels as the dependent variable, separating the explanatory variables into lipid and apolipoprotein components to avoid multicollinearity (Table 3). The magnitude of each factor's influence on serum sSDC1 levels was visualized using a heat map (Fig. 5).

**Table 3:** Multivariate analyses of factors affecting serum sSDC1 levels in patients with sepsis.

apoE phenotype	explanatory variables (mg/dL)	B	SE	β	t	*p*
Total (n = 259)	HDL-C	−9.853	1.952	−0.338	−5.049	<0.001
	LDL-C	0.365	1.160	0.020	0.315	0.753
	TG	−0.743	0.531	−0.090	−1.399	0.163
	apoAI	−10.017	1.542	−0.673	−6.494	<0.001
	apoAII	24.000	10.306	0.231	2.329	<0.05
	apoB	−2.611	1.517	−0.119	−1.722	0.086
	apoCII	−37.699	25.301	−0.142	−1.490	0.137
	apoCIII	79.150	14.678	0.542	5.393	<0.001
	apoE	−35.669	17.811	−0.132	−2.003	<0.05
apoE2/E3 (n = 235)	HDL-C	−6.292	1.231	−0.570	−5.110	<0.001
	LDL-C	−3.048	0.847	−0.288	−3.599	<0.001
	TG	1.001	0.376	0.308	2.666	<0.05
	apoAI	−7.220	0.865	−0.988	−8.346	<0.001
	apoAII	3.794	8.414	0.054	0.451	0.655
	apoB	−6.659	1.483	−0.459	−4.491	<0.001
	apoCII	45.600	21.950	0.246	2.077	<0.05
	apoCIII	24.078	11.775	0.201	2.045	<0.05
	apoE	22.166	12.083	0.166	1.834	0.075
apoE3/E3 (n = 176)	HDL-C	−8.631	2.716	−0.264	−3.178	<0.005
	LDL-C	1.594	1.521	0.085	1.048	0.296
	TG	0.172	0.433	0.031	0.398	0.691
	apoAI	−11.209	2.212	−0.710	−5.068	<0.001
	apoAII	32.962	14.059	0.308	2.344	<0.05
	apoB	−2.060	1.933	−0.096	−1.066	0.288
	apoCII	−72.370	35.047	−0.283	−2.065	<0.05
	apoCIII	91.991	19.757	0.695	4.656	<0.05
	apoE	−31.205	22.519	−0.121	−1.386	0.168
apoE3/E4 (n = 48)	HDL-C	−13.453	6.046	−0.367	−2.225	<0.05
	LDL-C	−2.967	3.113	−0.146	−0.953	0.346
	TG	−0.964	1.777	−0.089	−0.543	0.590
	apoAI	−10.190	5.315	−0.579	−1.917	0.062
	apoAII	15.583	33.290	0.143	0.468	0.642
	apoB	−4.418	4.327	−0.171	−1.021	0.313
	apoCII	15.841	63.875	0.048	0.248	0.805
	apoCIII	86.966	47.612	0.353	1.827	0.075
	apoE	−111.436	109.671	−0.168	−1.016	0.316

∗, To avoid multicollinearity, we performed a multiple regression analysis with serum sSDC1 levels as the dependent variable, separating the explanatory variables into lipid and apolipoprotein components.

HDL-C levels negatively influenced sSDC1 levels in all patients, irrespective of their apoE phenotype, with the most pronounced effect observed in those with apoE2/E3 (β = −0.570, *p* < 0.001). Similarly, although the effect was not statistically significant in patients with apoE3/E4 (*p* = 0.062), apoAI levels showed a negative association with sSDC1 levels in nearly all patients, with the strongest effect observed in those with apoE2/E3 (β = −0.988, *p* < 0.001). In contrast, to apoAI, the effect of apoAII on sSDC1 levels was less pronounced but still significant in patients with apoE3/E3 (β = 0.308, *p* < 0.05), where apoAII showed a positive association.

LDL-C and apoB levels were negatively associated with SDC1 levels, specifically in patients with apoE2/E3 (LDL-C, β = −0.288, *p* < 0.001; apoB, β = −0.459, *p* < 0.001), while TG levels were positively associated (β = 0.308, *p* < 0.05).

The relationship between apoCII and SDC1 levels varied by apoE phenotype: apoCII showed a positive association in patients with apoE2/E3 (β = 0.246, *p* < 0.05), but a negative association in those with apoE3/E3 (β = −0.283, *p* < 0.05). ApoCIII levels were positively associated with SDC1 in nearly all patients, with the strongest effect observed in the apoE3/E3 group (β = 0.695, *p* < 0.05). However, the association was not statistically significant in patients with apoE3/E4 (*p* = 0.075). Finally, although apoE levels showed no significant association with sSDC1 when analyzed by phenotype, a weak but statistically significant negative correlation was observed across all patients (β = −0.132, *p* < 0.05).

## 4 Discussion

Dyslipidemia is a well-known pathophysiological feature of sepsis and can influence its prognosis [2,3]. Consistent with previous studies, our data confirmed that patients with sepsis exhibited marked dyslipidemia, characterized by decreased levels of HDL and LDL, which predominantly consist of apoAI/apoAII and apoB, along with an increase in apoE-TRL. HDL is generated through de novo synthesis in the intestinal mucosa and liver, and is also formed during the catabolism of chylomicrons and possibly VLDL [31,32]. Notably, cell-surface SDC1 contributes to the metabolism of apoE-TRLs by acting as a co-receptor for LRP1 [10‒13].

As demonstrated in several previous studies [[33], [34], [35]], patients with sepsis show a marked increase in serum sSDC1 levels, suggesting that enhanced shedding of the extracellular domain of SDC1 may reflect infection-induced tissue damage driven by a systemic inflammatory response. Based on these previous findings, our findings raise the possibility that enhanced shedding of SDC1 may result from impaired catabolism and subsequent accumulation of apoE-TRLs, contributing to increased TG and decreased HDL levels. While elevated serum sSDC1 levels could partly reflect reduced renal clearance in septic patients with acute kidney injury, the distinct correlation patterns observed across apoE phenotypes in our study argue against renal dysfunction being the sole determinant. This hypothesis is further supported by our findings that sSDC1 levels in patients with sepsis were significantly inversely correlated with HDL-C, apoAI, and apoAII levels, and showed a tendency toward a positive correlation with TG levels.

Unlike the relationship between HDL-C and apoAI, the marked reduction in LDL-C without a significant decrease in apoB, along with the lack of a significant negative correlation between sSDC1 and apoB, may indicate that a possible reduction in apoB production was counterbalanced by impaired VLDL clearance or altered conversion to LDL, potentially due to inflammation [36]. Notably, the correlation between sSDC1 and LDL-C showed an opposite pattern: a positive correlation in control subjects and a negative correlation in patients with sepsis. A previous review suggested that impaired conversion of VLDL to LDL leads to reduced plasma LDL-C levels, even in the presence of high VLDL production, owing to decreased conversion of LDL from VLDL precursors [37]. Based on this evidence, our findings suggest that augmented SDC1 shedding may attenuate VLDL catabolism, impairing the conversion of VLDL to LDL.

Its anti-inflammatory properties are one of the pleiotropic functions of HDL [38]. However, inflammatory conditions induce structural and compositional modifications in HDL, including a reduction in its major apolipoprotein, apoA-I [39], decreased cholesterol ester content, and increased free cholesterol and TG levels. These alterations are primarily due to the suppressed activity of lecithin-cholesterol acyltransferase, ultimately impairing the anti-inflammatory function of HDL [39,40]. Taken together, hypo-HDL cholesterolemia in patients with sepsis may be influenced not only by a secondary reduction in HDL production due to impaired TRL catabolism, as mentioned above, but also by structural and compositional modifications of HDL induced by systemic inflammation.

ApoE levels were found to be significantly elevated in patients with sepsis, possibly reflecting the accumulation of apoE-TRL remnants in the bloodstream. As previously described, systemic inflammation may induce apoE upregulation [24]. Although the exact mechanism underlying this elevation remains unclear, interactions between apoE and its receptors are known to play a pivotal role in the apoE-TRL metabolism. Since these interactions are isoform-specific and influenced by apoE polymorphisms, particularly involving LDLR, LRP1, and possibly SDC1 [9,41], further analyses focusing on apoE polymorphisms were conducted.

ApoE3 and apoE2 are well-recognized for their anti-inflammatory properties and protective effects on endothelial function, whereas apoE4 is associated with proinflammatory activity and endothelial dysfunction [27,28,42]. Consistent with previous findings [34], our results showed a significantly higher frequency of the apoE4 allele in patients with sepsis, suggesting its potential role as a risk factor for sepsis exacerbation.

Sepsis-induced hypocholesterolemia, particularly reduced HDL-C levels, was more pronounced in patients with apoE3/E3. A negative correlation between sSDC1 and TC was prominent in apoE3/E4 patients, whereas a negative correlation between sSDC1 and HDL-C was evident in apoE2/E3 patients. A distinct negative correlation between sSDC1 and LDL-C levels was observed in patients with apoE3/E4. Although seemingly complex, these findings from our stratified analysis suggest that apoE plays a multifaceted role in regulating lipoprotein metabolism, modulating inflammatory responses, and maintaining endothelial integrity. In this context, hypocholesterolemia in patients with apoE3/E3 may be attributed primarily to metabolic alterations, whereas in patients with the apoE3/E4 phenotype, it may also reflect the detrimental effects of apoE4 on endothelial cells. Although the mechanism underlying the stronger correlation between sSDC1 with LDL-C rather than with HDL-C in patients with apoE3/E4 remains unclear, one possible explanation is the lower distribution of apoE4 on HDL particles compared to apoE3 or apoE2 [43]. Further studies are required to confirm this hypothesis.

The significant negative correlation between sSDC1 and HDL-C, together with the positive correlation between sSDC1 and TG, explicitly observed in patients with apoE2/E3, supports the hypothesis that reduced HDL-C may result from impaired catabolism of apoE-TRLs due to enhanced SDC1 shedding. This is consistent with the fact that apoE2 has an inherently low affinity for LDLR, the primary receptor responsible for the catabolism of apoE-TRLs, leading to their accumulation [9].

Consistent with the negative correlation between sSDC1 and HDL-C, patients with apoE2/E3 showed the strongest negative correlation between sSDC1 and apoAI, an important component of HDL. In contrast, a negative correlation between sSDC1 and apoAII, another component of HDL, was observed in patients with apoE3/E3 and apoE3/E4 but not in those with apoE2/E3. Although apoAI-rich HDL plays a protective role in atherosclerosis through cholesterol efflux, anti-inflammatory activity, and antioxidant effects [44], apoAII-rich HDL may promote atherosclerosis [45]. Although the precise mechanisms underlying these associations are unclear, our findings suggest that patients with apoE2/E3 may exhibit a more proinflammatory lipid profile than those with other apoE phenotypes. While no significant correlation between sSDC1 and apoB was observed in patients with sepsis, as described above, stratification by apoE phenotype revealed a significant negative correlation in patients with apoE3/E4, consistent with the negative correlation between sSDC1 and LDL-C.

ApoCII and apoCIII are recognized as an activator and an inhibitor of lipoprotein lipase (LPL), respectively [46]. The positive correlation between sSDC1 and apoCII in patients with apoE2/E3 and the negative correlation with apoCIII in patients with apoE3/E4 suggests that the respective upregulation of apoCII and downregulation of apoCIII may represent compensatory responses to impaired TRL catabolism via LPL. Although we did not observe significant differences in absolute TG levels between phenotypes, possibly due to the large variability inherent in septic cohorts, these correlation patterns imply phenotype-specific metabolic adaptations at the level of LPL regulation.

No correlation between sSDC1 and apoE was observed in patients with sepsis; however, stratification by apoE phenotype unexpectedly revealed a significant negative correlation in those with apoE3/E4. Although no explanation is currently available for these findings, reduced serum apoE levels in patients carrying apoE4 may serve as a potential indicator of sepsis exacerbation.

Finally, the apoE phenotype-specific determinants affecting serum sSDC1 levels were explored using multivariate analyses. Consistent with the results described above, HDL-C and apoAI levels were negatively associated with sSDC1 levels in all patients, with the most substantial effect observed in patients with apoE2/E3. These findings suggest that reducing anti-inflammatory apoAI-rich HDLs may exacerbate inflammation and promote SDC1 shedding. In contrast, an increase in proinflammatory apoAII-rich HDLs is associated with sSDC1 levels in patients with apoE3/E3, but not in those with apoE2/E3. This suggests that apoAII-rich HDL may play a more prominent role in SDC1 shedding in individuals with apoE3/E3, indicating isoform-specific differences in lipoprotein function and the inflammatory response. The apoE phenotype may influence the balance between apoAI- and apoAII-rich HDLs, thereby affecting systemic inflammation and endothelial injury.

Correlation analysis revealed inverse associations between sSDC1 and LDL-C or apoB levels, specifically in patients with apoE3/E4. These associations were not observed in multivariate analysis, suggesting potential confounding effects. In contrast, LDL-C and apoB levels emerged as independent predictors of sSDC1 levels in patients with apoE2/E3, indicating a direct link between atherogenic lipoproteins and endothelial injury in this subgroup. The apoE4 may exert broader detrimental effects on endothelial function, such as promoting inflammation, oxidative stress, and vascular permeability, which may contribute to elevated sSDC1 levels independent of atherogenic lipoproteins. Similarly, the multivariate model did not confirm the association between sSDC1 and apoCIII observed in the correlation analysis for patients with apoE3/E4, likely because of similar confounding effects. Conversely, multivariate analysis revealed a significant positive association between sSDC1 and apoCIII, particularly in patients with apoE3/E3, suggesting that apoCIII directly promotes SDC1 shedding under septic conditions. ApoCIII has proinflammatory properties and interferes with TRL clearance, which may exacerbate endothelial dysfunction [47]. These mechanisms may enhance endothelial damage and SDC1 shedding in patients with apoE3/E3, possibly owing to less efficient TRL clearance than in apoE2 carriers.

Notably, the effect of apoCII on sSDC1 levels appeared to differ according to the apoE phenotype, showing a positive association in patients with apoE2/E3 and a negative association in patients with apoE3/E3. These findings may reflect the isoform-specific effects of apoE on LPL-mediated TRL metabolism. In patients with apoE2/E3, impaired TRL clearance due to the low LDLR affinity of apoE2 [9] may lead to compensatory upregulation of apoCII to enhance LPL-mediated lipolysis, potentially protecting against endothelial injury and SDC1 shedding. However, efficient TRL clearance in patients with apoE3/E3 [9] may reduce the need for apoCII, while excess apoCII could promote lipid accumulation, LPL saturation, or inflammation, thereby exacerbating endothelial dysfunction and SDC1 shedding.

This study has several strengths. Comprehensive analyses stratified by apoE polymorphisms allowed us to identify isoform-specific alterations in lipoprotein metabolism and their associations with inflammatory and endothelial injury markers in sepsis. Our findings highlight a potential link between increased SDC1 shedding and altered apoE-TRL catabolism, offering new insights into sepsis-associated dyslipidemia. However, several limitations must be acknowledged. First, due to the retrospective nature of the study design and limited access to detailed clinical records, we were unable to obtain comprehensive data regarding potential confounding factors. Specifically, information on the use of lipid-lowering medications (e.g., statins and fibrates), history of dyslipidemia, and specific markers of renal function (e.g., eGFR) was missing. Since sSDC1 is partially cleared by the kidneys, renal impairment could theoretically elevate serum sSDC1 levels independently of syndecan shedding. However, the observation of phenotype-specific correlations suggests a biological link beyond simple renal accumulation. Second, the precise time lag between sepsis onset and blood sampling varied, which may have contributed to the heterogeneity of the data. Third, the small sample size, especially in the apoE2/E3 and apoE3/E4 subgroups, may limit the statistical power and generalizability. Consequently, our findings should be considered preliminary and hypothesis-generating. Future prospective studies with rigorous adjustment for these clinical variables are warranted to validate the mechanisms suggested here.

In conclusion, our findings suggest that serum sSDC1 levels are associated with alterations in lipid and apolipoprotein profiles in sepsis, and that these associations may differ according to apoE phenotype. These observations provide clinical insight into the relationship between inflammation-related sSDC1 dynamics and lipid/apolipoprotein remodeling in sepsis.

## Availability of data and materials

The data that support the findings of this study are openly available from the corresponding author, [KY], upon reasonable request.

## Ethical approval and consent to participate

The present study was approved by the Ethical Review Board of Shinshu University School of Medicine (approval number, 5762). Written informed consent was obtained from all patients.

## Funding

This research was supported by a Grant-in-Aid for Scientific Research from the 10.13039/501100001691Japan Society for the Promotion of Science (JSPS KAKENHI Grant Number 24K10594 to K.Y.).

## CRediT authorship contribution statement

**Riho Shimizu:** Conceptualization, Data curation, Formal analysis, Investigation, Methodology, Writing – original draft. **Shogo Akahane:** Data curation, Formal analysis, Investigation, Methodology. **Harue Suzuki:** Investigation, Methodology. **Hiroto Matsuura:** Investigation, Methodology. **Yui Uematsu:** Investigation, Methodology. **Wakana Iimura:** Investigation, Methodology. **Nau Ishimine:** Conceptualization, Investigation, Methodology, Resources. **Kazuyoshi Yamauchi:** Conceptualization, Data curation, Formal analysis, Funding acquisition, Investigation, Methodology, Project administration, Resources, Software, Supervision, Validation, Visualization, Writing – original draft, Writing – review & editing.

## Declaration of competing interest

The authors declare that they have no known competing financial interests or personal relationships that could have appeared to influence the work reported in this paper.

## Data Availability

Data will be made available on request.
